# Human Polycomb 2 Protein Is a SUMO E3 Ligase and Alleviates Substrate-Induced Inhibition of Cystathionine β-Synthase Sumoylation

**DOI:** 10.1371/journal.pone.0004032

**Published:** 2008-12-24

**Authors:** Nitish Agrawal, Ruma Banerjee

**Affiliations:** Department of Biological Chemistry, School of Medicine, University of Michigan, Ann Arbor, Michigan, United States of America; Auburn University, United States of America

## Abstract

Human cystathionine β-synthase (CBS) catalyzes the first irreversible step in the transsulfuration pathway and commits homocysteine to the synthesis of cysteine. Mutations in CBS are the most common cause of severe hereditary hyperhomocysteinemia. A yeast two-hybrid approach to screen for proteins that interact with CBS had previously identified several components of the sumoylation pathway and resulted in the demonstration that CBS is a substrate for sumoylation. In this study, we demonstrate that sumoylation of CBS is enhanced in the presence of human polycomb group protein 2 (hPc2), an interacting partner that was identified in the initial yeast two-hybrid screen. When the substrates for CBS, homocysteine and serine for cystathionine generation and homocysteine and cysteine for H_2_S generation, are added to the sumoylation mixture, they inhibit the sumoylation reaction, but only in the absence of hPc2. Similarly, the product of the CBS reaction, cystathionine, inhibits sumoylation in the absence of hPc2. Sumoylation in turn decreases CBS activity by ∼28% in the absence of hPc2 and by 70% in its presence. Based on these results, we conclude that hPc2 serves as a SUMO E3 ligase for CBS, increasing the efficiency of sumoylation. We also demonstrate that γ-cystathionase, the second enzyme in the transsulfuration pathway is a substrate for sumoylation under in vitro conditions. We speculate that the role of this modification may be for nuclear localization of the cysteine-generating pathway under conditions where nuclear glutathione demand is high.

## Introduction

Cystathionine β-synthase (CBS)^1^ catalyzes the pyridoxal 5′-phosphate-dependent condensation of serine and homocysteine to form cystathionine, which represents the first committed step in the transsulfuration pathway for cysteine synthesis [1], [2]. Cystathionine is then converted to cysteine in a reaction catalyzed by γ-cystathionase (CSE). Gene disruption of CSE leads to marked hypertension and impaired vasorelaxation, confirming the importance of this enzyme as a source of the gaseous signaling molecule, H_2_S, which is formed as a side reaction, presumably from cysteine [3]. Deficiency of CBS activity is the most common cause of hereditary hyperhomocysteinemia [4] and over one hundred patient mutations in CBS have been described [5]. Curiously, a subset of pathogenic CBS mutations when mimicked in vitro exhibit no apparent biochemical penalty, and in fact, sometimes display higher activity than wild-type enzyme [6], [7]. This has led us to suggest the hypothesis that these mutations may disrupt interactions between CBS and other proteins, which are important for its cellular functions. In an effort to identify such interacting partner proteins, a yeast two-hybrid screen was undertaken and furnished a disproportionate number of proteins related to the sumoylation pathway including the SUMO (small ubiquitin-like modifier) conjugation enzyme Ubc9 (ubiquitin-conjugating enzyme), the SUMO ligases, PIAS1 (protein inhibitor of activated STAT1) and PIAS3, the RanGTPase binding protein, RanBP, and human polycomb group protein 2, hPc2 [8]. Human CBS was subsequently shown to be a target for sumoylation both in vitro as well as in vivo [8].

SUMO is a small ubiquitin-related modifier protein, which is covalently attached to target proteins. The human genome encodes three functional isozymes of SUMO: SUMO-1, -2 and -3 that are expressed ubiquitously, whereas the paralog, SUMO-4, may not be fully processed and exhibits a more restricted tissue distribution [9], [10]. Posttranslational modification by SUMO is one mechanism for dynamic regulation of target proteins and elicits diverse effects including subcellular relocalization typically to the nucleus, changes in protein partner interactions and modulation of the DNA-binding and/or transactivation activities of transcription factors [11].

The coordinated activities of SUMO activating, conjugating and ligating enzymes are required for sumoylation, a process that has parallels with ubiquitination. The first step in this mechanism is catalyzed by the E1 ubiquitin-activating heterodimeric enzyme, Aos1-Uba2, and results in the formation of a thioester bond between the C-terminal glycine residue in SUMO and a cysteine residue in Uba2. The next step involves transfer of SUMO to the active site cysteine residue in the E2 ubiquitin carrier protein, Ubc9. In the final step, the activated carboxyl group of the terminal glycine residue in SUMO is transferred to the ε-amino group of a lysine residue in the target protein to form an isopeptide bond. This step is often facilitated by an E3 ubiquitin-protein isopeptide ligase but some targets are efficiently sumoylated by E2 alone under *in vitro* conditions [11].

The mechanism by which E3 ligases enhance the kinetics, specificity and/or efficiency of Ubc9-mediated sumoylation remains unclear, and they might be particularly important for SUMO conjugation to substrate proteins that contain variant consensus motifs [12]. The canonical SUMO acceptor site contains a lysine residue in the sequence motif, ΨKXE/D, in which Ψ is a hydrophobic residue and X is any amino acid. However, lysines in variant or non-canonical sequences can also be sumoylated [12]. E3 proteins facilitate SUMO ligation either by binding to substrate proteins or by binding to the E2 conjugation enzyme, Ubc9, and stimulating transfer of SUMO to the substrate or to another SUMO molecule in case of modifier chain formation [13]. To date, four groups of E3 ligases have been identified: the SP-RING ligases, which includes the PIAS family proteins [14], the nuclear pore protein, Ran BP2 [15], the human polycomb group member, hPc2 [16] and the histone deacetylase, HDAC4 [17].

The polycomb group proteins are well-studied epigenetic regulators of the fly homeotic gene cluster that function as silencers and help maintain the transcriptional history of target genes over many cell generations [18]. In mammals, homologs of these proteins are important for maintaining stem cell pluripotency and the identity of differentiated cells [19]. hPc2 is a member of the polycomb repressive complex 1, which is required for stable maintenance of transcriptional repression [18], [20], [21], [22]. hPc2 and its relatives are characterized by an N-terminal chromatin organization modifier (chromo) domain and a C-terminal C-box [23]. hPc2 functions as a SUMO E3 ligase for at least four target proteins including the transcriptional co-repressor, CtBP [24], the transcriptional regulator, SIP1 [25], the homeodomain-interacting protein kinase 2, HIPK2 [26], and the DNA methyltransferase, Dnmt3a [27].

In this study, we demonstrate that hPc2 functions as an E3 ligase for human CBS and enhances its sumoylation by SUMO-1. Sumoylation and catalytic turnover appear to be competitive since sumoylation is inhibited in the presence of the CBS substrates, serine and homocysteine that generate cystathionine and homocysteine and cysteine that generate H_2_S as well as the product, cystathionine. Substrate and product-induced inhibition of sumoylation is averted in the presence of hPc2. Conversely, sumoylation of CBS inhibits its activity, and this effect is enhanced in the presence of hPc2, which increases the extent of CBS sumoylation. We also demonstrate that the second enzyme in the transsulfuration pathway, γ-cystathionase (CSE), is a substrate for sumoylation under in vitro conditions.

## Results

### Sumoylation of CBS is enhanced by hPc2 under in vitro conditions

**Figure 1:**
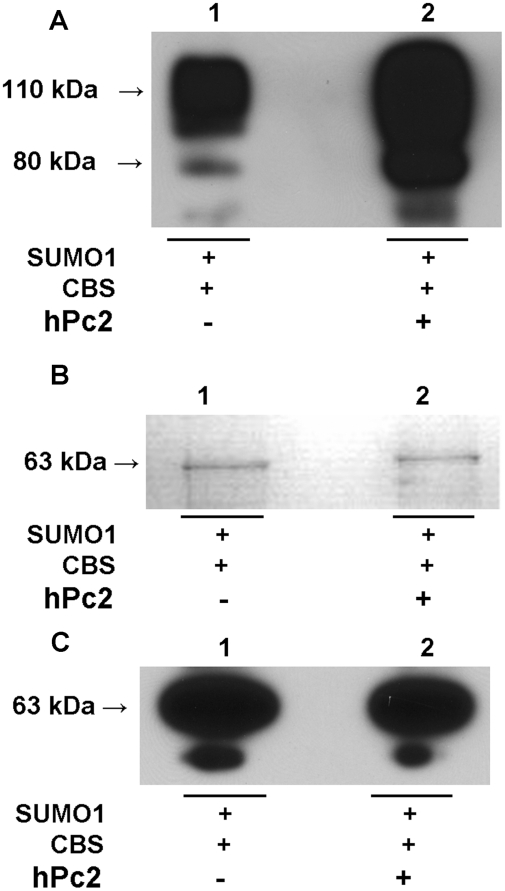
Effect of hPc2 on sumoylation of human CBS. A. The in vitro sumoylation reaction was performed as described under Methods and SUMO-1 antibody was used to detect the presence of sumoylated CBS. Lanes 1 and 2 show sumoylation reactions that were conducted in the absence (lane 1) and presence (lane 2) of hPc2. B and C. Equal loading controls for CBS in the two reaction mixtures. The CBS concentration prior to the start of the sumoylation reaction was identical in both lanes as detected by Coomassie blue staining (B) or by Western blot analysis using CBS antibody (C).

To evaluate whether hPc2 serves as an E3 ligase for sumoylation of CBS, the SUMO modification reaction was reconstituted *in vitro* in the presence or absence of hPc2. Western blot analysis using a SUMO-1 antibody revealed the presence of two high molecular mass bands (Figure 1) corresponding to ∼80 kDa and ∼110 kDa respectively as seen previously [8]. However, in contrast to the earlier in vitro sumoylation reactions in which an aliquot of rabbit reticulocyte lysate was added [8], the current kit-based assay contained only the purified component proteins and exhibited a higher efficiency of modification. The presence of catalytic amounts of hPc2 increased the efficiency of the sumoylation reaction (Figure 1).

### Effect of CBS substrates on sumoylation

**Figure 2:**
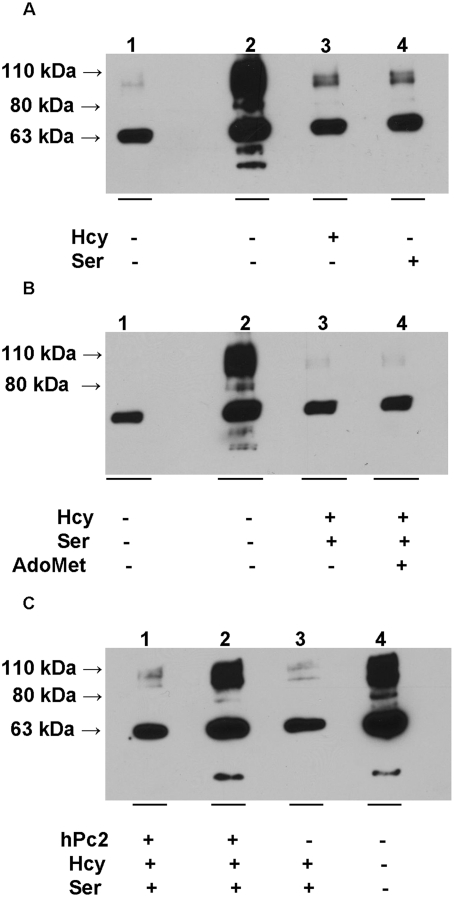
Effect of substrates on the sumoylation of human CBS. Western blot analysis using CBS-antibody of sumoylation reaction mixtures containing the substrates for CBS in the absence (A) or presence (B) of the allosteric activator, S-adenosylmethionine (AdoMet). (C) The effect of hPc2 on sumoylation of human CBS in the presence of substrates.

To investigate whether the sumoylation of CBS was affected by the presence of substrates, the in vitro sumoylation assays were performed in the presence or absence of the substrates, homocysteine and serine. The presence of either substrate inhibited the sumoylation reaction and only the 110 kDa band was visible under these conditions (Figure 2A). The presence of the allosteric activator, S-adenosylmethionine, had no further effect on the efficiency of CBS sumoylation (Figure 2B). Substrate-induced inhibition of sumoylation was avoided by the presence hPc2 (Figure 2C).

**Figure 3:**
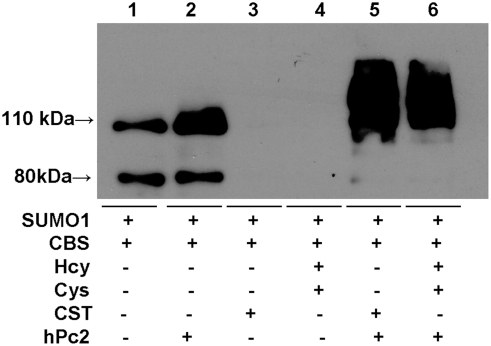
Effect of the H_2_S generating substrates, cysteine and homocysteine, and the CBS reaction product, cystathionine, on sumoylation in the presence and absence of hPc2. The Western blot was detected using SUMO-1 antibody.

We next tested the effect of the product, cystathionine, formed by the condensation of serine and homocysteine, on sumoylation of CBS. Again, sumoylation was completely inhibited in the presence of 10 mM cystathionine and this inhibition was alleviated by the presence of hPC2 (Figure 3). Similarly, the substrates for H_2_S generation by CBS, homocysteine and cysteine [28], inhibit sumoylation but only in the absence of hPc2 (Figure 3). We note that the total intensity is consistently higher in lanes showing CBS sumoylation regardless of whether SUMO-1 (Figures 1A and 3) or CBS (Figure 2) antibody is employed for detection, despite equal loading of CBS in each lane (Figure 1B,C). The basis for this is not understood.

### Effect of sumoylation on CBS activity

The effect of sumoylation on CBS activity was assessed following a 6 h sumoylation reaction in the presence or absence of hPc2. As controls, the activity of CBS incubated for the same duration but in the absence of the sumoylating agents or in the presence of only hPc2 was determined. In comparison to the controls, sumoylation resulted in the loss of 28% and 70% of CBS activity in the absence and presence respectively, of hPc2. We note that CBS exists in a mixed oligomeric state ranging from a dimer to higher order oligomers [29]. In the in vitro assay, CBS subunits are hypersumoylated resulting in a mixture of unsumoylated subunits and those with 1 or more SUMO modifications (Figure 2A and B, lane 2 and Figure 2C, lanes 2 and 4). Hence, although the efficiency of CBS monomer sumoylation in the in vitro assay is low (20–50%), the extent of inhibition exceeds the level of sumoylation suggesting that modification of some of the subunits interferes with the activity of the native protein.

### In vitro sumoylation of CSE

While the significance of nuclear localization of CBS is not known, it is pertinent to ask if the next enzyme in the pathway, CSE, is subjected to sumoylation and therefore also has the potential to relocate to the nucleus under some conditions. As a first step towards addressing this question, we have examined whether recombinant human CSE can be sumoylated. Under in vitro conditions, we observe sumoylation of CSE and the appearance of two major bands with molecular masses of ∼65 and 85 kDa versus 45 kDa for the parent CSE subunit. The presence of 5 mM cystathionine, the substrate for CSE, did not affect the efficiency of the sumoylation reaction.

## Discussion

In mammals, the transsulfuration pathway plays important roles in clearing homocysteine, in methionine homeostasis, and in providing cysteine especially in cells that exhibit a high turnover of the antioxidant, glutathione. CBS is a heavily regulated enzyme at the homocysteine metabolic junction and defects in CBS result in severely elevated homocysteine levels and multiorgan deficits, whose molecular basis is poorly understood. A yeast two-hybrid analysis conducted in our laboratory identified a number of proteins involved in the sumoylation pathway as potential interacting partners of CBS including a number of E3 ligases e.g. PIAS1, PIAS3 and hPc2 [8]. It was speculated that the multiplicity of E3 ligases identified by the yeast two-hybrid approach could have been a consequence of complex formation between the E3 prey and the endogenous yeast E2, which in turn interacted with the CBS bait. The PIAS proteins are members of the largest family of E3 ligases whereas hPc2 has a much more limited range of substrates possibly because of its nuclear localization in polycomb bodies [30]. In this study, we have examined the effect of hPc2 on the efficiency of CBS sumoylation.

**Figure 4:**
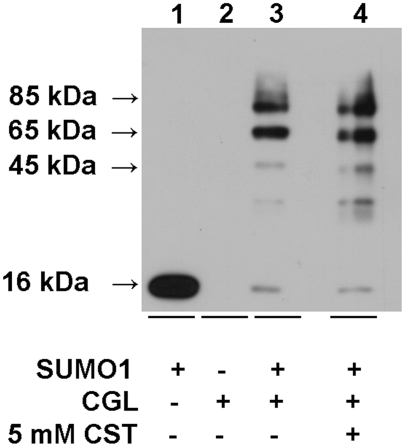
Human CSE is a target of in vitro sumoylation. Sumoylation of CSE was conducted as described under Methods in the presence or absence of 5 mM cystathionine. The Western blot was detected using SUMO antibody. The band with a molecular mass of ∼16 kDa represents SUMO-1 present in the reaction mixture.

**Figure 5:**
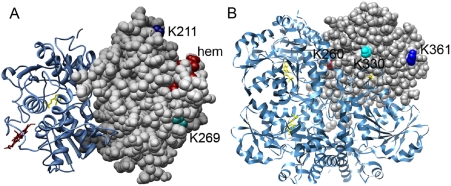
Structures of human CBS and CSE showing locations of potential SUMO modification sites. A. Locations of K211 and K269 in the canonical and noncanonical SUMO modification sites respectively in human CBS (PDB 1M54). The two subunits of CBS are shown in grey and blue and the heme and PLP cofactors are shown in red and yellow respectively. K211 and K269 are shown in one of the two subunits in navy and cyan respectively. B. Locations of K361 (blue), K330 (cyan) and K260 (red) in one of the four subunits of human CSE (PDB file 2NMP). The PLP cofactor is shown in yellow in ball and stick representation.

Both SUMO-2 and SUMO-3 have internal consensus SUMO modification sites that allow polymerization on target proteins [13], [31]. In contrast, an internal sumoylation site is absent in SUMO-1 and ligation of only monomeric units is observed with this modifier. Since SUMO-1 was used in the assay mixtures, the observation of multiple bands corresponding to sumoylated CBS in the vitro assay (Figure 1A), which has also been reported previously [8], and to CSE (Figure 4) suggests that sumoylation occurred at more than one site. Although SUMO is an ∼11 kDa protein, modification of the target proteins typically results in an ∼20–30 kDa band shift [32]. The subunit molecular mass of human CBS is 63 kDa and the 80 kDa band appears to be associated with sumoylation at K211, which resides within a canonical sumoylation site. Mutation of K211 to arginine leads to loss of the 80 kDa band [8]. Human CBS has a variant sumoylation site, LK_269_EK, in which the acidic residue found in the consensus motif is missing. K269 like K211, is relatively surface exposed in the structure of the truncated CBS dimer (Figure 5A). Human CSE has at least three lysine residues in potential sumoylation motifs of which one, LK_361_ND, is in a consensus sequence whereas two, LK_330_NL and LK_260_TL, are present in what appear to be variant sequences. Of these, K260 seems unlikely to be a sumoylation site based on the crystal structure as it is buried in the subunit interface while the other two lysines are relatively surface exposed (Figure 5B). Examples of sumoylation at variant sites have been reported, and while it is not known how Ubc9 recognizes such target sequences, it is speculated that the E3 component may be especially influential in modifications involving variant sites [12], [33]. We note however, that the SUMO hypermodification of CBS that is observed in the in vitro assay is not seen in vivo where only the 80 kDa modified band is seen [8].

In the absence of hPc2, sumoylation and substrate binding to CBS appear to be competitive. Hence, the presence of either serine or homocysteine, cysteine and homocysteine or the product, cystathionine, results in a markedly reduced efficiency of SUMO modification (Figures 2 and 3). This suggests that substrate binding induces a conformational change that reduces the efficiency of interaction between Ubc9 and CBS. While the K_M_ for homocysteine (5 mM [34]) and cysteine (6 mM [28]) are high compared to the intracellular concentrations of homocysteine and cysteine in mammalian cells, which is in the 10–100 µM range, the intracellular concentration of serine is significantly higher (∼560 µM) [28] while the K_D_ for serine for human CBS is low (7.5 µM [35]). This suggests that in the cytoplasm, sumoylation of CBS may be largely inhibited under most circumstances.

Although the majority of SUMO targets are nuclear proteins, a growing number of non-nuclear targets in the cytoplasm, plasma membrane and mitochondria have been identified [9]. Sumoylation of CBS could trigger its nuclear localization. Enhanced sumoylation of CBS in the presence of hPc2 might be expected to occur in the nucleus since hPc2 has a restricted subnuclear localization within polycomb bodies. We note however that initial nuclear localization studies on CBS have revealed its presence in the nuclear scaffold [8]. We also note that most SUMO targets do not exhibit quantitative modification. Rather, a small proportion of the target protein tends to be modified and low-level sumoylation can have significant cellular effects as described for transcription factors and for thymine DNA glycosylase [9].

The significance of SUMO modification of CBS is not known nor is the role of this protein in the nuclear compartment. Similarly, it is not known whether CSE is sumoylated in the cell and whether some proportion of this enzyme is found in the nucleus. One possibility is that the nuclear CBS moonlights in an altogether different role, which remains to be identified. An alternative is that sumoylation leads both enzymes of the transsulfuration pathway into the nucleus where they function locally in the removal of homocysteine, in the provision of cysteine and/or H_2_S, a gaseous signaling molecule. Of these, cysteine production may be the most significant since this amino acid is the limiting reagent in the synthesis of glutathione. The nuclear∶cytoplasmic ratio of glutathione varies from ∼1 to 4 depending on the cell cycle stage and the nuclear pool peaks just prior to exponential cell growth [36]. It is estimated that between 4 to 8 percent of glutathione synthesis occurs in the nucleus and activities for the biosynthetic enzymes, γ-glutamyl cysteine ligase and glutathione synthetase, have been reported in this compartment [37]. Peak telomerase activity is correlated with high nuclear glutathione levels [38], and it is believed that a reducing environment might be important for the optimal functioning of other nuclear proteins. CBS activity also varies with the stage of cell-growth with maximal expression occurring during cell proliferation [39]. Hence, nuclear localization of the transsulfuration pathway enzymes, CBS and CSE, might be a strategy for ensuring local delivery of cysteine under conditions of high glutathione demand in the nucleus.

A potential problem with this model for SUMO-dependent translocation of the transsulfuration pathway enzymes is that sumoylation of CBS is associated with inhibition of its activity (Table 1). Hence, if sumoylation is required to bring CBS (and potentially, CSE) into the nucleus, desumoylation of nuclear CBS would be needed for the enzyme to be active. We note that SUMO modification is dynamic and the modified targets can be rapidly desumoylated by isopeptidases that are efficient at removing SUMO. CBS is a long-lived protein with a half life that is estimated to be ∼49 h [40]. Hence, once it is translocated into the nucleus and desumoylated, it could potentially function as a catalyst in this compartment for some time.

**Table 1:** Sumoylation inhibits CBS activity.

Additions^a^	Pre-incubation time	Activity µmol h^−1^ mg^−1^	Percent activity remaining^b^
CBS	none	286±36	
CBS	6 h	157±21	100
CBS+hPc2	6 h	180±11	100
CBS+SUMO-1^a^	6 h	121±25	72
CBS+SUMO-1+hPc2^a^	6 h	50±13	30

a In vitro sumoylation of CBS was performed as described under Methods.

b The activity remaining after 6 h of incubation needed for the sumoylation reaction is reported relative to the average of the CBS and CBS+hPc2 assay mixtures (169 µmol h^−1^ mg^−1^).

It is interesting to note that a component of the well-studied cytoplasmic folate cycle has been demonstrated to have a nuclear localization that is dependent on SUMO modification [41]. Cytoplasmic serine hydroxymethyltransferase is a target of SUMO modification both in vivo and in vitro and has been shown to also have a nuclear residence. The two other enzymes needed for de novo thymidylate synthesis, dihydrofolate reductase and thymidylate synthase, also contain SUMO modification consensus sequences and sumoylation might underlie the nuclear localization of this part of the folate-dependent one-carbon pathway for local synthesis of the DNA precursor, thymidylate [41].

In summary, we have demonstrated that both enzymes in the transsulfuration pathway, CBS and γ-cystathionase, are targets of in vitro sumoylation and that hPc2 functions as an E3 ligase for CBS. It is not known if the physiological relevance of this modification is to relocate some proportion of the transsulfuration pathway into the nucleus for local production of cysteine needed for glutathione synthesis under cellular conditions or if these proteins serve a different function in the nuclear compartment.

## Materials and Methods

### Materials

The following materials were purchased from Sigma: D,L-homocysteine, L-cysteine, serine and cystathionine. Glutathione S-transferase was purchased from GE Healthcare. ECL anti-rabbit IgG horseradish peroxidase-linked whole antibody (from donkey), ECL anti-mouse IgG, horseradish peroxidase-linked whole antibody (from sheep) and [^14^C]-serine (specific activity = 1.64 mCi/mmol (50 µCi/vial)) were purchased from GE Healthcare. Aos1/ Uba2, Ubc9, SUMO-1 and SUMO-1 rabbit monoclonal antibody were purchased with the SUMOlink kit from Active Motif, Carlsbad, CA. Chicken polyclonal CBS antibody, previously generated in our laboratory, was used for CBS Western blot analyses. Horseradish peroxidase-labeled goat anti-chicken IgY was purchased from Aves Labs, Oregon. Detection of horseradish peroxidase conjugated secondary antibodies was performed using the supersignal chemiluminescent substrate purchased from Pierce Biotechnology, IL. Protease Inhibitor tablets were purchased from Roche Diagnostics, Indianapolis, IN, USA.

### CBS, CSE and hPc2 Protein Expression and Purification

Recombinant human CSE was purified as described previously [42]. Recombinant human CBS was expressed and purified using the parent plasmids pGEX4-T1/hCBS, which produces a fusion protein with glutathionine S-transferase at the N-terminus, as described previously [43] with the exception that the initial anion exchange chromatography step was omitted. *E. coli* BL21 (DE3) cells were transformed with the expression vector for human hPc2 (pHis-Parallel1 encoding amino acids 2–529), which was a generous gift from Dr. David Wotton (University of Virginia). Transformed BL21 (DE3) was grown in Luria Bertaini medium at 37°C till the OD_600_ reached 0.6. IPTG (isopropyl β-D-1-thiogalactopyranoside) was added to a final concentration of 5 µM and the culture was grown for an additional 12 h at 18°C. The cells were harvested by centrifugation at 6000×g for 30 min and washed with lysis buffer (50 mM sodium phosphate, pH 8.0, 300 mM NaCl and 10% v/v glycerol) and resuspended in 100 ml of lysis buffer supplemented with 10 mM 2-mercaptoethanol, 1 µg/ml leupeptin and 1 mM PMSF. The cells were disrupted by sonication and the extract was centrifuged at 16,000×g for 30 min. The supernatant was loaded onto a Ni-NTA column (8×3 cm) pre-equilibrated with lysis buffer and washed with the lysis buffer supplemented with 10 mM 2-mercaptoethanol and 20 mM imidazole. The column was eluted with a gradient ranging from 20–250 mM imidazole in lysis buffer containing containing 10 mM 2-mercaptoethanol. Approximately 3 mg of hPc2 was obtained per liter of culture by this method and was stored at −80°C until further use.

### In vitro sumoylation of CBS and CSE

Sumoylation reactions were performed using the commercially available SUMOlink kit as follows. Aos1/Uba2, Ubc9 (2 µl each) and SUMO-1 (10 pmol, 2 µl) were added to either recombinant human CBS (8 pmol) or recombinant human CSE (12 pmol) in the presence or absence of purified hPc2 (2 pmol) in the commercially supplied sumoylation buffer to a final volume of 22.5 µl. When the effect of CBS substrates on the efficiency of sumoylation was examined, the reaction mixture contained one or more of the following additions: 0.38 mM AdoMet, 30 mM L-serine, 15 mM DL-homocysteine, 10 mM cystathionine, 10 mM cysteine. When the effect of substrate on the efficiency of CSE sumoylation was tested, 5 mM cystathionine was added. The reaction mixture was incubated for 6 h at 37°C and quenched with an equal volume of 5×SDS loading buffer (250 mM Tris HCl pH 6.8, 10% SDS, 30% glycerol, 5% β-mercaptoethanol, 0.02% bromophenol blue) followed by incubation at 95°C for 10 min. Each experiment was performed at least in triplicate.

### Western blot analysis

To identify the presence of SUMO-modified CBS or CSE, the reaction mixtures were separated on a 12% polyacrylamide gel under denaturing conditions. The gels were transferred to polyvinylidene fluoride microporous membranes (Biorad, Hercules, CA) at room temperature for 4 h at 350 mA using a Transblot apparatus (BioRad). After blocking, the membranes were probed with polyclonal CBS antibody(1∶2000 dilution) or monoclonal SUMO-1 antibody (1∶4000 dilution). The recombinant hPc2 used in these studies contains an N–terminal His tag and was detected with an anti-His tag antibody (1∶3000 dilution). CBS and SUMO-1 Western blots were detected with horseradish peroxidase-labeled goat anti chicken antibody (1∶50,000 dilution for CBS) and ECL anti-rabbit IgG horseradish peroxidase-linked whole antibody from donkey (1∶ 50,000 dilution for SUMO-1) respectively. Western blots for hPc2 were detected with mouse IgG horseradish peroxidase-linked whole antibody from sheep (1∶50,000 dilution). The membranes were visualized using the super signal chemiluminescent substrate.

### Effect of sumoylation on CBS activity

CBS activity was measured in the radiolabeled assay described previously [43]. To test the effect of sumoylation on activity, CBS (1 µg) was sumoylated as described above except that the amount of CBS and SUMO-1 in the assay mixture were doubled (to 16 pmole of CBS subunits and 20 pmol of SUMO-1) and incubated for 6 h at 37°C. At the end of this incubation, 5 µl of each reaction mixture was removed and quenched with an equal volume of SDS loading buffer as described above and to the remaining volume, the following assay components were added in a final volume of 200 µl: 250 mM Tris HCl, pH 8.6, 0.25 mM PLP, 0.38 mM AdoMet, 30 mM [^14^C]-serine (∼50,000 dpm/µmol), and the sumoylated mixture and incubated for 5 min at 37°C. The CBS reaction was started with 30 mM D,L-homocysteine, incubated at 37°C for 60 min and terminated with 200 µl of 10% trifluoroacetic acid. The mixture was centrifuged for 10 min at 11,000×*g* to remove any precipitate and the sample was processed as described previously [43].
